# Effect of the Micronization of Pulp Fibers on the Properties of Green Composites

**DOI:** 10.3390/molecules26185594

**Published:** 2021-09-15

**Authors:** Bruno F. A. Valente, Armando J. D. Silvestre, Carlos Pascoal Neto, Carla Vilela, Carmen S. R. Freire

**Affiliations:** 1 CICECO—Aveiro Institute of Materials, Department of Chemistry, University of Aveiro, Campus Universitário de Santiago, 3810-193 Aveiro, Portugal; bfav@ua.pt (B.F.A.V.); armsil@ua.pt (A.J.D.S.); cvilela@ua.pt (C.V.); 2 RAIZ—Research Institute of Forest and Paper, The Navigator Company, Rua José Estevão, 3800-783 Eixo, Portugal; Carlos.Neto@thenavigatorcompany.com

**Keywords:** poly(lactic acid), poly(hydroxybutyrate), cellulose fibers, micronization, green composites

## Abstract

Green composites, composed of bio-based matrices and natural fibers, are a sustainable alternative for composites based on conventional thermoplastics and glass fibers. In this work, micronized bleached Eucalyptus kraft pulp (BEKP) fibers were used as reinforcement in biopolymeric matrices, namely poly(lactic acid) (PLA) and poly(hydroxybutyrate) (PHB). The influence of the load and aspect ratio of the mechanically treated microfibers on the morphology, water uptake, melt flowability, and mechanical and thermal properties of the green composites were investigated. Increasing fiber loads raised the tensile and flexural moduli as well as the tensile strength of the composites, while decreasing their elongation at the break and melt flow rate. The reduced aspect ratio of the micronized fibers (in the range from 11.0 to 28.9) improved their embedment in the matrices, particularly for PHB, leading to superior mechanical performance and lower water uptake when compared with the composites with non-micronized pulp fibers. The overall results show that micronization is a simple and sustainable alternative for conventional chemical treatments in the manufacturing of entirely bio-based composites.

## 1. Introduction

The increasing demand for eco-friendly materials associated with the implementation of legislation and policies towards a more sustainable society has triggered the replacement of synthetic and petrochemical-based materials with bio-based ones [1,2]. In the field of composite materials, as far as reinforcements are concerned, a notorious increase in the use of natural-based fibers in replacement of synthetic counterparts, such as glass or aramid, has been witnessed in the last decade. Several natural fibers such as flax, hemp, jute, kenaf, wood flour, or pulp have been thoroughly investigated [3,4]. In fact, the market of natural fiber-based composites, also commonly referred to as biocomposites, is already established (USD 22.3 billion in 2019) and some large companies, such as Stora Enso, UPM, and Sappi, have launched over the years a range of products composed of conventional fossil-based and non-biodegradable thermoplastics, such as polypropylene (PP) and polyethylene (PE), reinforced with cellulosic fibers [5,6,7,8].

PP and PE, together with other polymeric matrices, such as poly(vinyl chloride) (PVC), polystyrene (PS), and acrylonitrile butadiene styrene (ABS), are still the main thermoplastics used in the biocomposite industry [9]. However, and despite the clear environmental benefits over composites reinforced with synthetic fibers, biocomposites whose matrices are derived from fossil resources still pose some environmental threats. Specifically, the non-renewability and non-biodegradability of the matrices as well as the unfeasibility to recycle the composites are still their major drawbacks [10,11]. In this regard, the logical alternative is to manufacture fully bio-based composites by replacing the non-biodegradable petrochemical-derived matrices with bio-based polymers, the so-called bioplastics [12].

Poly(lactic acid) (PLA) and poly(hydroxybutyrates) (PHB) are among the few bioplastics currently produced at a commercial scale. They have comparable properties to some commodity plastics, can be processed with technologies applied to conventional thermoplastics, and, because of the increasing demand for bioplastics and maturing of production technologies, their prices are becoming more affordable [10,13,14]. The ever-increasing number of studies regarding the use of bioplastics in the composite field reflect the growing interest on these sustainable polymers [4]. However, these so-called green composites, for which both the matrix and reinforcement are bio-based, face some of the same challenges of their petroleum-based counterparts. Although the interfacial adhesion between for instance PLA and cellulose fibers is naturally stronger than for many other thermoplastic polymers, the lack of compatibility between the hydrophilic cellulose fibers and the hydrophobic matrices is still an issue [11,15]. The intrinsic hydrophilicity and high aspect ratio of the cellulosic materials often lead to agglomeration and poor dispersion of the fibers in the polymeric matrices [16]. Therefore, the visual aspect of the composites is inevitably impaired, as well as their mechanical performance. The strategies commonly used for composites with petrochemically based matrices have also been investigated for PLA and PHB-based counterparts to overcome these challenges and further increase the overall performance of such materials. Pre-treatments of the fibers, such as alkali treatments [17] or surface modifications including, for example, acetylation [18] or silylation [19], are among the most common. Other compatibilization strategies, such as the use of coupling agents [20,21,22], which can be done prior to or during melt-mixing, have also been tested. From the industrial point of view, however, the manufacturing process should be as simple, inexpensive, and efficient as possible. Thus, as an alternative to the aforementioned chemical methods, mechanical procedures may also be efficient to overcome some drawbacks. In this sense, size reduction by milling processes, such as pan milling [23], ball milling [24,25], or shear and cooling milling [26], may be used to decrease the length and width of the fibers to the micro or nano range. For instance, ball milling has been already used to reduce the size of bleached pine kraft pulp to particles sizes inferior to 120 µm [24,25]. The results showed that size reduction was an efficient method to increase the dispersion of cellulose materials in PLA or PLA/poly(3-hydroxybutyrate-*co*-3-hydroxyvalerate) (PHBV) based composites. In addition, the size reduced particles still had a reinforcing effect as proved by the increased mechanical properties. However, upon these mechanical treatments, the fibers lost their fibrillar morphology, becoming irregular particles instead. Additionally, the aspect ratio and crystallinity index (CI) of the fibers drastically decreased [24,25].

In this context, the present study aims at manufacturing fully green composites using bio-based matrices, namely PLA and PHB, reinforced with micronized bleached Eucalyptus kraft pulp (BEKP). Micronization is proposed as a mechanical treatment to reduce both the length and width of the fibers to the micrometric range, without compromising their fibrillar morphology and crystallinity but rather improving their dispersion within the polymeric matrices. This strategy has the additional advantage of being practical and free of any solvents or chemicals. The influence of the fiber load and effect of their aspect ratio were studied. The composites were evaluated regarding their interfacial morphology, mechanical performance, water uptake capacity, melt flowability, and thermal properties.

## 2. Results and Discussion

**Figure 1:**
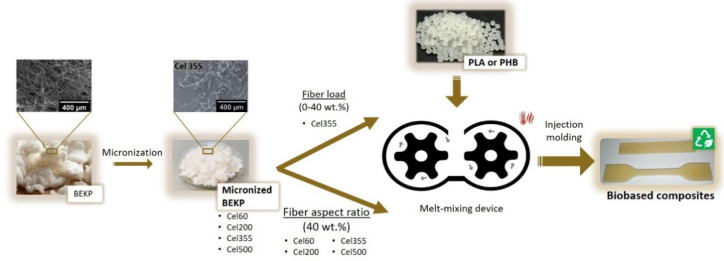
Schematic illustration of the experimental procedure used in the present study.

In the present work, the impact of the micronized fiber load and the effect of their aspect ratio on the properties of green composites with two distinct grades of both PLA and PHB matrices were evaluated. The four bio-based polymeric matrices (i.e., PLA 3D860, PLA 3100HP, PHB P209E, and PHB P226) were selected according to their mechanical properties, melt flow index, and recommended uses [27,28]. To assess the impact of the micronized fibers’ load, Cel355 fibers with an intermediate aspect ratio (26.6) were melt-mixed with the thermoplastic matrices. To study the effect of the aspect ratio of the fibers, four micronized fibers with distinct aspect ratios and BEKP (Table 1) were also melt-mixed with all four thermoplastic matrices for a fixed fiber load of 40 wt.% (Figure 1).

**Figure 2:**
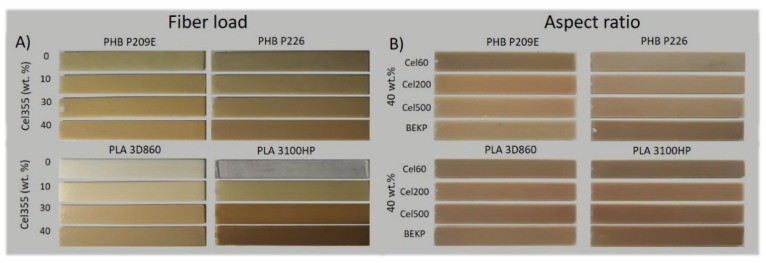
Digital photographs of the PHB- and PLA-based composites with: (**A**) different Cel355 loads (0, 10, 30 and 40 wt.%), and (**B**) with micronized fibers with distinct aspect ratios and non-micronized BEKP fibers.

The macroscopic aspect of the composites specimens after the injection molding is shown in Figure 2. The composites with different loads of Cel355 (Figure 2A) are increasingly darker with the increment of the fiber load, particularly for the composites with PLA. This behavior can be attributed to the high temperatures used during the processing of these composites, particularly during injection molding (195 °C), which led to some thermal degradation of cellulose. A similar observation was reported by Ozyhar et al. [29] for composites of PLA reinforced with 40 wt.% of wood fibers, where the increase in the composites color intensity was also associated to some thermal degradation of the polysaccharides during processing. Besides the increased color intensity, no visible agglomerates of the fibers could be perceived for composites with different reinforcement loads or for those with 40 wt.% micronized fibers with distinct aspect ratios (Figure 2B), which might indicate that the fibers were homogeneously dispersed in the matrices. The density values of the polymeric matrices agreed with the specification of the products [27,28] (Table S1). As for the composites, as expected, an increase on the fiber load raised the density of the composite, given the superior density of the fibers [30] in comparison with the thermoplastic polymers used in the present work. Conversely, the density of the composites with fibers having different aspect ratios remained relatively unchanged (Table S1). Then, the morphology, mechanical properties, water-uptake capacity, flowability, and thermal stability of all the composites were evaluated.

### 2.1. Morphology

**Figure 3:**
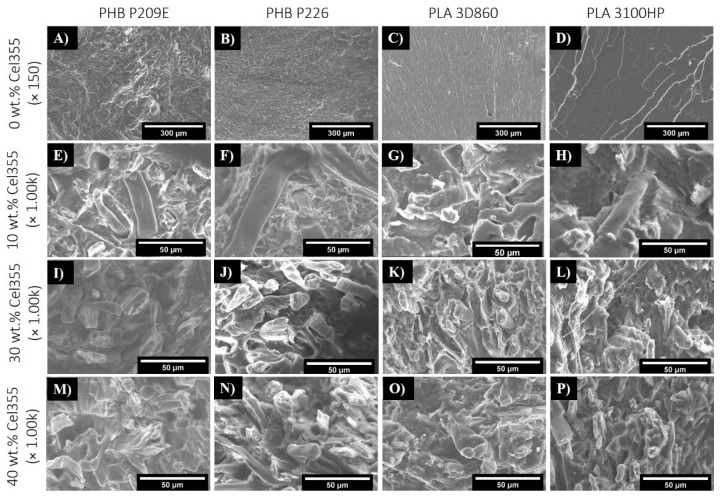
SEM micrographs of the fractured surfaces of (**A**–**D**) the neat PHB and PLA matrices and of (**E**–**P**) the corresponding composites reinforced with different Cel355 loads: (**E**–**H**) 10 wt.%, (**I**–**L**) 30 wt.% and (**M**–**P**) 40 wt.%.

**Figure 4:**
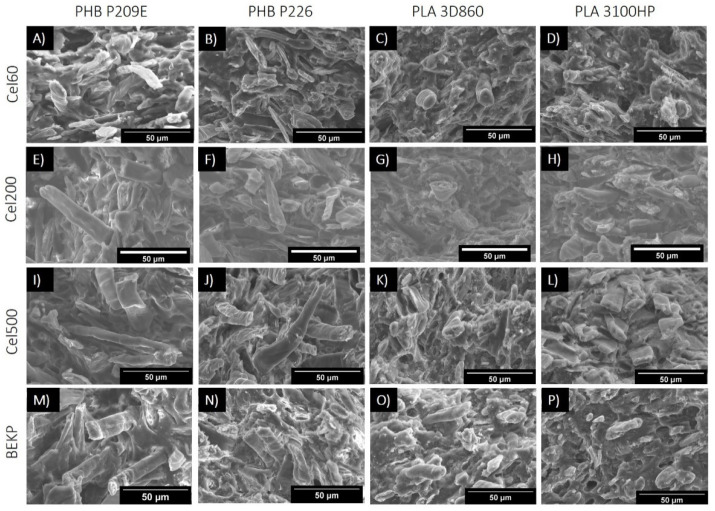
SEM micrographs of the fractured surfaces of composites with fibers having different aspect ratios: (**A**–**D**) Cel60; (**E**–**H**) Cel200; (**I**–**L**) Cel500 and (**M**–**P**) BEKP.

The mechanical properties of composites are highly dependent on the ability to efficiently transfer energy from the polymeric matrix to the fibers that in turn depends on the dispersion of the fibers and on the interfacial adhesion between them and the matrix [31]. The cross-section fracture surfaces of the neat matrices, obtained after tensile tests, and of the composites with different loads of Cel355 are displayed in Figure 3, while those of the composites with micronized fibers with different aspect ratios are shown in Figure 4. Despite the strong tendency of the hydrophilic cellulose fibers to stack together when compounded with hydrophobic thermoplastic matrices [31], no visible agglomerates or bundles of micronized fibers can be observed on the micrographs of the composites with different contents of Cel355 (Figure 3) or with fibers with different aspects ratios (Figure 4). This is a confirmation of the good dispersion of the micronized fibers in the PLA and PHB matrices, which agrees with the visual observation of the test specimens (Figure 2)**.**

Besides this good dispersion, the fracture surfaces of the composites also revealed the existence of some fibers pull-outs and voids (Figure 3E, top). This phenomenon has been extensively described in the literature and is usually attributed to the different phobic nature of the constituents [32,33,34]. However, due to the carboxylic and hydroxyl end groups of PHB and PLA, there is a high degree of compatibility between these matrices and cellulose fibers when compared with common thermoplastic matrices, such as PP or PE [15,35]. The fact that fractured fibers are more prevalent on the micrographs than the pulled-out fibers is also evidence of such a compatibility. Additionally, the micrographs point to a slightly better compatibility of the fibers with PLAs than with the PHBs, which may be related to the chemical structural differences between these two polyesters, with PHBs having a longer monomeric chain.

Concerning the composites loaded with different micronized fibers (Figure 4), as the fiber aspect ratio increases, alongside with the length, the fiber pullouts and voids created during the tensile testing are more frequent and the pulled-out fibers are longer, as would be expected. In regard to the interfaces between the fibers and matrices, although only minor differences can be observed on the micrographs, shorter micronized fibers may have improved dispersion and embedment over the longer and higher aspect ratio non-treated BEKP. Such difference of the effect of the fiber dimensions on the interfacial morphology was described by Madyan et al. [36], in which larger fibers led to bigger gaps and cracks, and smaller fibers were better imbedded in the matrix.

### 2.2. Mechanical Properties

#### 2.2.1. Tensile Properties

**Figure 5:**
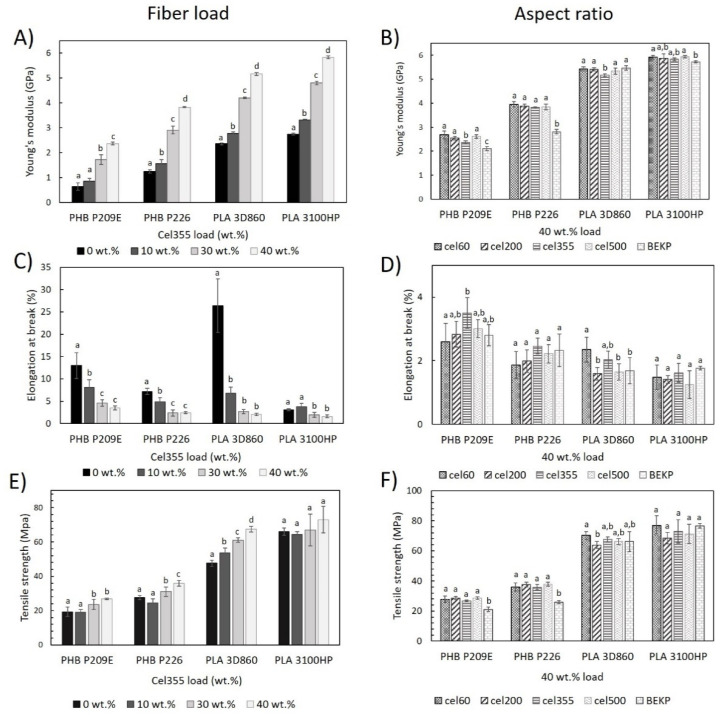
Tensile properties of the PHB and PLA-based composites (**A**,**C**,**E**) reinforced with different loads of Cel355 and (**B**,**D**,**F**) reinforced with fibers having different aspect ratios for a load of 40 wt.%. Different letters (a,b,c,d) indicate statistically significant differences (*p*  <  0.05).

The Young’s modulus, tensile strength, and elongation at break of the polymeric matrices and of the corresponding composites are presented in Figure 5. The results show, for both PLA- and PHB-based composites, a gradual increment in the Young’s modulus with the increasing Cel355 fiber load, which is in agreement with previous findings on the effect of the fiber load, and can be easily explained by the higher stiffness of the cellulose fibers compared to the polymeric matrices [37]. Specifically, the Young’s modulus of composites reinforced with 40 wt.% Cel355 increased by 1.7 GPa (2.37 ± 0.06 GPa) and 2.6 GPa (3.83 ± 0.02 GPa) in comparison with the respective PHB matrices (PHB P209E: 0.64 ± 0.02 GPa; PHB P226: 1.25 ± 0.07 GPa), and by 2.8 GPa (5.16 ± 0.05 GPa) and 3.1 GPa (5.83 ± 0.06 GPa) compared to the corresponding PLA matrices (PLA 3D860: 2.40 ± 0.04 GPa; PLA 3100HP: 2.75 ± 0.04 GPa). As the biggest increases are noted for the PLA-based composites, those findings corroborate the relatively better compatibility of the fiber with PLAs than with PHBs, as previously discussed.

In previous studies of PLA reinforced with a 40 wt.% load of bleached softwood kraft pulp, a similar increase of 3.0 GPa was registered when compared to the matrix [38], which shows that the micronized fibers used in this work, despite their reduced sizes, still have a good reinforcing effect. The effectiveness of such a reinforcing effect is closely related to the fibrillar morphology and crystallinity (Table 1) of the micronized fibers since, unlike other mechanical treatments (e.g., ball milling), the micronization of the fibers still retained their fibrillar morphology and only a slight decrease in the crystallinity was observed [24,25]. Moreover, the Young’s modulus of the composites of PHBs reinforced with 40 wt.% Cel355 were within the range of the commercial products based on PP or PE reinforced with 40 wt.% pulp fibers (1.9 to 4.6 GPa) and the Young’s modulus of the PLA-based composites were clearly superior to those of the mentioned commercial products [5,6,7].

Concerning the effect of the aspect ratio on the Young’s modulus, for the most part, changes in the micronized fiber aspect ratio (in the range between 11.0 and 28.9) had little influence on this parameter (Figure 5B). Qiang et al. studied the effect of size variations of ball-milled bleached pine kraft pulp in composites with PLA or binary mixtures of PLA and poly(3-hydroxybutyrate-*co*-3-hydroxyvalerate) (PHBV), and also concluded that the loading content contributes more to the variation of the mechanical properties than the fiber aspect ratio [24,25]. Interestingly, PHB-based composites reinforced with micronized fibers had a significantly superior Young’s modulus than the composites reinforced with BEKP. In fact, the average Young’s modulus of the PHB composites with the micronized fibers is superior to the corresponding composites with BEKP by 26% for PHB P209E and 29% for PHB P226.

With respect to the elongation at break, as expected, a reduction in this parameter was generally observed with the increasing fibers content [10]. All composites with 40 wt.% reinforcement had elongation at break values below 3.5% (Figure 5C), which is inferior to those of the commercial products mentioned before (4.0 to 6.5%) [5,6,7]. In contrast, with only a few exceptions, the aspect ratio of the fibers did not have a significant effect on the elongation at break of the composites, as portrayed in Figure 5D.

The representation of the tensile strength of the composites as a function of the fiber load (Figure 5E) shows that higher reinforcement contents of micronized fibers generally raised the tensile strength. For example, in composites with 40 wt.% Cel355, an improvement on the tensile strength of 39, 30, 42, and 10% was noted in comparison with the corresponding PHB P209E, PHB P226, PLA 3D860, and PLA 3100HP matrices. The present results contradict some published works in which often the increase in the fiber load tended to decrease the tensile strength, either for PLA or PHB matrices [37,39]. Additionally, the tensile strength values of composites of PLA 3D860 (67.5 ± 1.7 MPa) and PLA 3100HP (72.9 ± 7.7 MPa) reinforced with 40 wt.% Cel355 were superior to those of commercial products of PP reinforced with pulp fibers (47 to 64 MPa) [5,6,7]. These results are certainly related to the relatively good dispersion of the micronized fibers within the polymeric matrices, as well as to their interfacial adhesion, as previously observed by SEM analysis. The composites with micronized fibers having different aspect ratios had similar tensile strengths (Figure 5F), which has also been previously reported for composites of PLA and mixtures of PLA and PHBV [24,25]. Despite the almost negligible effect of the aspect ratio, the tensile strength of all the PHB composites reinforced with the micronized fibers was far superior to those with BEKP, which agrees with the Young’s modulus trend previously described.

#### 2.2.2. Flexural Properties

**Figure 6:**
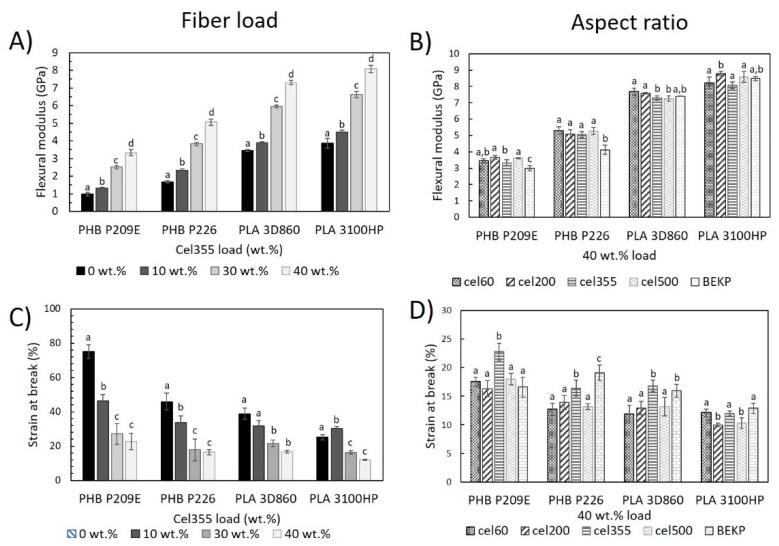
Flexural properties of) the PHB- and PLA-based composites reinforced (**A**,**C**) with different loads of Cel355 and (**B**,**D**) with fibers having different aspect ratios for a load of 40 wt.%. Different letters (a,b,c,d) indicate statistically significant differences (*p*  <  0.05).

The effect of the reinforcement of Cel355 fibers on the flexural modulus of composites based on PLA and PHB matrices was in line with the results of the Young’s modulus previously discussed, i.e., the increase in the fiber load led to a higher flexural modulus (Figure 6A). This is not surprising, though, given that the flexural modulus often follows the same pattern as the Young’s modulus [39,40]. Moreover, the results obtained for PHBs reinforced with 30 wt.% Cel355 (2.5 ± 0.1 GPa for PHB P209E and 3.8 ± 0.1 GPa for PHB P226) are similar or even better to those obtained by Gunning et al., in which PHB matrices were reinforced with 30 wt.% hemp (≈1.6 GPa), Lyocell (≈1.9 GPa) and Jute (≈3.8 GPa) fibers [31]. For PLA-based composites, the flexural modulus of the composites reinforced with 40 wt.% Cel355 (7.3 ± 0.1 GPa for PLA 3D860 and 8.1 ± 0.2 GPa for PLA 3100HP) was even superior to the values reported for PLA reinforced with 40 wt.% of sisal (≈6.2 GPa) [41], wood (≈4.5 GPa) [37], or with pulp fibers from poplar wood (≈5.7 GPa) [37]. On the contrary to the effect observed for the fiber load, the fiber aspect ratio had little influence on the flexural modulus of the composites. However, PHB-based composites reinforced with BEKP had an inferior modulus than the composites reinforced with micronized fibers (Figure 6B), which is also in agreement with the variation in the Young’s modulus. Such results are probably related to the larger dimensions of the BEKP that can lead to larger gaps and cracks, creating weak points for composites to fail [36]. On the contrary, the shorter micronized fibers may have improved distribution and embedment on the matrix, improving its reinforcing effect [42].

The strain at break, calculated as the ratio between the extension at break and the maximum deflection (20 mm), provides important information about the flexibility of the composites. As observed in Figure 6C, the polymeric matrices are more flexible than the corresponding composites and higher fiber content led to lower flexibility, which can be also credited to the high stiffness of the fibers in comparison with the matrices [10]. For example, the incorporation of only 10 wt.% of Cel355 in the most flexible matrix (PHB P209E) led to a reduction of the strain at break from 75.1 ± 4.1% to only 46.3 ± 4.9%. However, only small differences could be observed for the strain at break in the composites reinforced with fibers having different aspect ratios. The results of the strain at break are, in general, in agreement with the elongation at break determined on the tensile tests.

#### 2.2.3. Impact Properties

**Figure 7:**
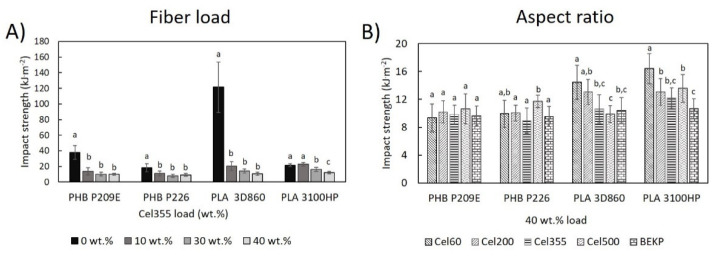
Impact properties of PHB- and PLA-based composites (**A**) reinforced with different loads of Cel355 and (**B**) reinforced with fibers having different aspect ratios for a load of 40 wt.%. Different letters (a,b,c) indicate statistically significant differences (*p*  <  0.05).

The impact strength of neat polymeric matrices and composites as a function of the fiber load and aspect ratio were evaluated following the Charpy edgewise impact test (Figure 7). Regarding the polymeric matrices, the impact strength of the unnotched specimens of the PHBs, which are 38.0 ± 8.7 kJ m^−2^ for PHB P209E and 18.2 ± 5.2 kJ m^−2^ for PHB P226, were within the range of impact strengths reported in the literature, which can vary from 5 kJ m^−2^ to over 65 kJ m^−2^ [43,44]. The impact strength of PLA 3100HP (21.5 ± 1.8 kJ m^−2^) was also near the values reported elsewhere [44,45]. However, the energy required to break an unnotched specimen of PLA 3D860 (121.4 ± 32.4 kJ m^−2^) was more than three times higher than for any PHB matrix studied and more than five times higher than for PLA 3100HP, which can be justified by the fact that PLA 3D860 is designated by the manufacturers as a high impact polymer [28].

The incorporation of micronized fibers (Cel355) significantly reduced the ability of the material to absorb the impact (Figure 7A). In fact, the most accentuated decreases were noted for composites with only 10 wt.%. of fibers. For higher cellulose contents, the decreases were not significantly different. In the literature, the effect of fiber incorporation in the impact strength of biocomposites is still unclear and contradictory. Previous studies have shown both increases and decreases in the impact strength upon incorporation of cellulosic fibers [46,47]. It’s known that many factors may influence the composite’s impact properties, such as the crystallinity and stiffness of the individual components. However, and despite the good homogeneity and dispersion of micronized fibers on the matrices, as observed in the SEM images (Figure 3), some defects on the interface may still be responsible for the deterioration of the impact strength [37,48]. Such defects on the interface, even at lower fiber loads, leads to crack initiation and propagation, which are responsible for the decreased amount of force the material can absorb during impact [32]. Nonetheless, the obtained values are in line with literature data. For instance, Oliver-Ortega et al. recorded an impact strength of 21.7 ± 1.2 kJ m^−2^ in composites of PLA reinforced with 10 wt.% bleached kraft softwood pulp [45], which is similar to the impact strength obtained for PLA 3D860 reinforced with 10 wt.% of Cel355 (20.4 ± 5.6 kJ m^−2^) and PLA 3100HP (22.8 ± 2.3 kJ m^−2^).

In Figure 7B, the results indicate that for PHB matrices, the changes in the aspect ratio of the fibers did not translate into different impact strength properties. In contrast, for composites based on PLA, smaller aspect ratio fibers seemed to favor the impact strength. The relatively better interfacial adhesion between the PLA matrices and fibers, as seen by SEM, combined with their smaller aspect ratio, reduced the formation of defects on the composite, consequently lowering sites for initiation and propagation of cracks [36]. In comparison with other materials, the obtained impact properties of composites with a fiber load of 40 wt.%, ranging from 9.0 ± 1.8 kJ m^−2^ to 16.4 ± 2.2 kJ m^−2^, are inferior to those of the commercial products of PP or PE reinforced with 40 wt.% pulp fibers (33 to 42 kJ m^−2^) [5,6,7], which still presents a challenge.

### 2.3. Water Uptake Capacity

**Figure 8:**
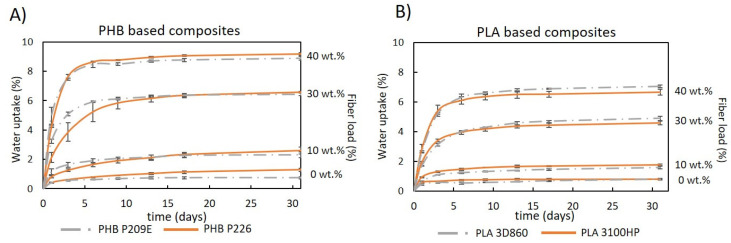
Water uptake as function of time for: (**A**) PHB- and (**B**) PLA-based composites reinforced with Cel355.

The evaluation of the water absorption of the composites reinforced with natural fibers is of enormous importance because it is normally associated with dimensional stability issues and a decrease of the mechanical properties [49]. The water absorption of the neat PLA and PHB matrices used in this study were under 1.3 ± 0.1%, after 31 days of immersion (Figure 8), which is in accordance with the values reported in the literature for PLA [40] and PHB [50] matrices. Since cellulose has high affinity for water [51,52], all composites showed higher water absorptions that increased with the growing fiber contents, which is not surprising given there is unanimity in the fact that increasing the content of the hydrophilic portion of the composites leads to an increased water absorption [22,45]. Moreover, all composites followed the same uptake pattern: a rapid increase during the first few days of immersion and stabilization after reaching saturation [53]. It is also noticeable that composites with PHBs had higher water-uptakes than composites with PLAs. This observation can also be related to the apparent inferior compatibility between the fibers and PHBs, as previously discussed (Figure 3), which leads to enhanced water penetration thought the composite material [45,51]. These current findings contradict the observations made by Yatigala et al., in which wood fiber-reinforced PLA composites had higher-water uptakes (11.9%) than the PHB counterpart (10.2%) [22]. However, and more importantly, the composites prepared in this work with 30 wt.% of the micronized fiber (Cel355) had significantly inferior water-uptakes (6.4 ± 0.1% to 6.6 ± 0.1% for PHBs and 4.6 ± 0.1% to 4.9 ± 0.1% for PLAs) than the ones previously reported by the authors for the same reinforcement percentage [22].

**Figure 9:**
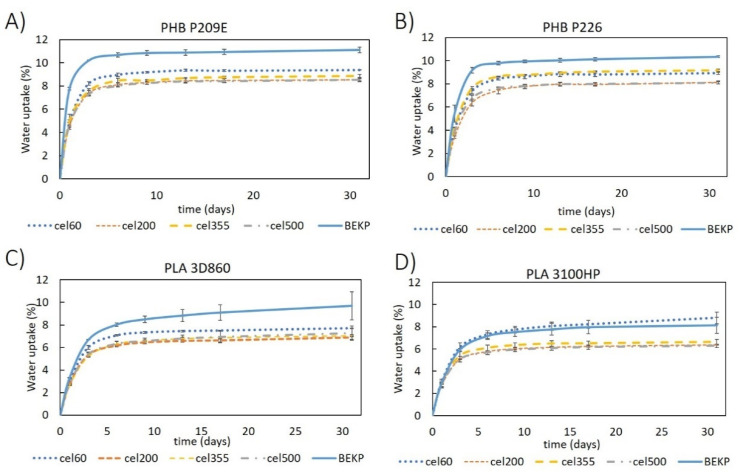
Water uptake as function of time for composites prepared with different micronized cellulose samples for a fiber load of 40 wt.% in matrices of: (**A**) PHB P209E; (**B**) PHB P226; (**C**) PLA 3D860 and (**D**) PLA 3100HP.

Interestingly, composites reinforced with micronized fibers have a clear advantage over the ones reinforced with non-micronized BEKP (Figure 9). Apart from those of PLA 3100HP, composites with BEKP not only have higher water-uptake values (up to 11.1 ± 0.2%) but also higher water absorption rates during the first days, mainly for composites with PHBs. The positive effect of micronization on the water-uptake behavior is in line with the results of the tensile and flexural moduli, which strengthens the idea that the reduced size of micronized fibers favors their embedment in the matrix, preventing water from permeating easily into the composite [51]. In reference to the aspect ratio of the micronized fibers (11.0 to 28.9), with the exception of composites based on PLA 3100HP, all composites with different micronized fibers had similar water-uptake values.

### 2.4. Melt Flow Rate

**Figure 10:**
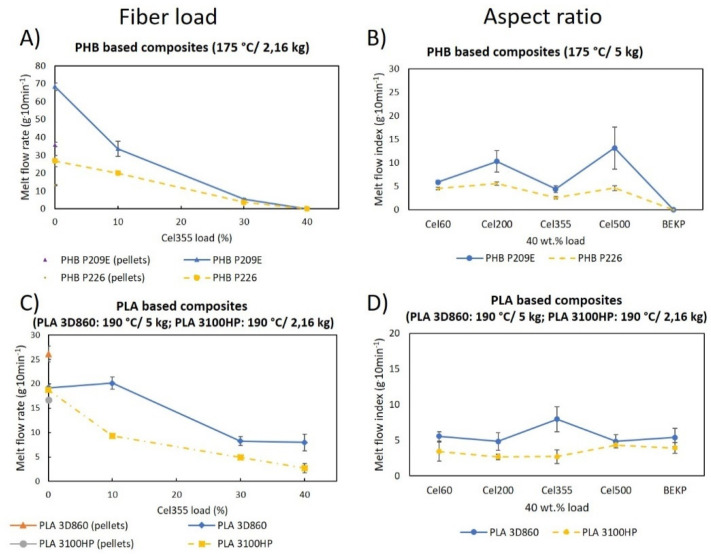
MFR of the polymeric matrices before and after melt-mixing: (**A**,**C**) their composites with Cel355 and (**B**,**D**) of the composites with fibers having different aspect ratios.

The melt flow rate measures the ease of a molten thermoplastic material to flow under very specific conditions, such as the diameter and length of the die and the cylinder [54]. The outcome is expressed in grams of the material that flows over the course of ten minutes when standard weights are applied at a predetermined temperature [54]. Figure 10A,C represent the melt flow rate of the PLA and PHB matrices prior to and after melt-mixing, along with the melt flow rate of the composites with different fiber loadings, while Figure 10B,D show the melt flow of the composites having fibers with different aspect ratios.

According to Figure 10A,C, the melt flow rate of the polymeric matrices increased after being submitted to the melt-mixing procedure, except for PLA 3D860, probably due to the presence of additives in this PLA sample. The effect is more pronounced for the PHBs, with increments of 90% for PHB P209E and a 103% increase for PHB P226. Giving that there is an inversely proportional relationship between the MFR and the molecular weight of the thermoplastic polymers, the increase in the MFR is most likely due to some thermal degradation during the melt-mixing procedure, which certainly leads to the decrease in the molecular weight of the polymeric matrices [55]. This is supported by previous studies as Carrasco F. et al., who studied the influence of melt processing on the molecular weight and melt flowability of PLA, concluding that the thermal degradation caused by melt processing decreased the molecular weight of PLA from 212.3 kDa to 162.5 kDa. As a direct consequence, the melt flow rate increased from 7.0 g·10 min^−1^ to 10.7 g·10 min^−1^ [56]. A similar outcome was verified for PHB matrices where melt processing decreased the molecular weight from 535 × 10^3^ g·mol^−1^ to 208 × 10^3^ g·mol^−1^, leading the MFR to raise from 19 g·10 min^−1^ to 26 g·10 min^−1^ [57].

When the micronized fibers (Cel355) were added to the matrices, the MFR gradually decreased. Similar observations have been reported for composites of PLA or PHB reinforced with different natural fibers (e.g., jute and hemp) [31,58] and the decrease was mainly attributed to the bad dispersion of the fibers within the matrices and to the fiber/fiber and fiber/matrix frictions [31,58]. However, since the composites in the present work show a good dispersion of the micronized fibers, as previously discussed, the decrease in the MFR probably has to do with the increase in the friction between fibers and between fibers with the matrices, which its turn decreases the ease of flow [58]. Moreover, the most pronounced decreases in the MFR were noted for PHB-based composites, which, according to the SEM micrographs, were slightly less compatible with the fibers than the PLA matrices. The poorer interfacial adhesion may increase the friction between the fibers and the matrices, leading in turn to a decreased flowability of the composite material [59].

The fiber aspect ratio had little effect on the MFR, especially for PLA-based composites. However, for the composites based on PHBs, the micronization had a positive effect on the MFR. All the composites reinforced with micronized fibers showed MFRs between 4.5 ± 0.3 g·10 min^−1^ and 13.2 ± 4.5 g·10 min^−1^, while those reinforced with BEKP did not flow at all. In composites with PHBs, where the compatibility with fibers is slightly inferior than with PLAs, as previously observed by SEM (Figure 3 and Figure 4), the BEKP fibers, which are considerably longer and wider than the micronized fibers, may lead to a higher fiber entanglement, preventing the composite material from flowing [31,60]. In contrast, the shorter micronized fibers with improved dispersion and embedment may have prevented fiber entanglement. Such an observation emphasizes that the micronization of the BEKP fibers is a useful strategy to improve the flowability of the composites, which is of great importance for injection molding applications to ensure proper mold-filling and good quality of the materials [31,61].

### 2.5. Thermal Analysis

**Figure 11:**
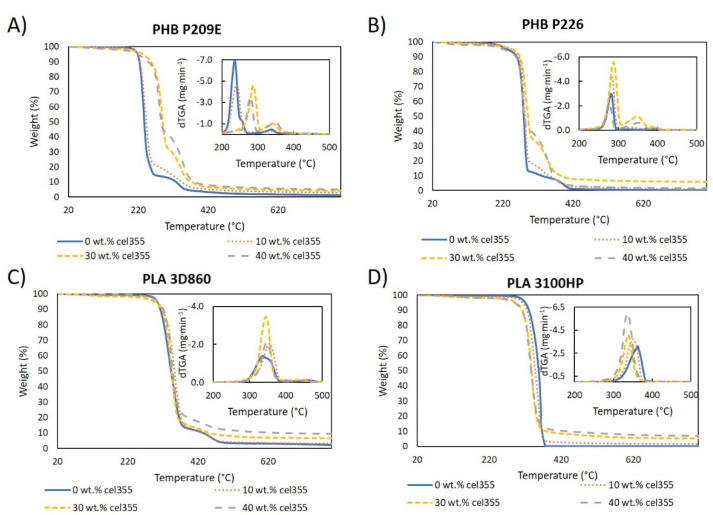
Thermogravimetric and derivative (dTGA) curves of the pure polymeric matrices and composites with different loads of Cel355 (10, 30, and 40 wt.%) in matrices of: (**A**) PHB P209E; (**B**) PHB P226; (**C**) PLA 3D860 and (**D**) PLA 3100HP.

The thermal stability of all the thermoplastic polymers and composites were studied by thermogravimetric analysis (TGA). The thermogravimetric curves of PLA and PHB matrices, as well as of the corresponding composites with different fiber loads, are shown in Figure 11 and those of the composites with fibers having different aspect ratios are represented in Figure S5. The neat polymeric matrices showed a degradation profile with one major weight loss at 236 °C and 281 °C for PHB P209E and PHB P226, respectively, and 336 °C and 364 °C for PLA 3D860 and PLA 3100HP, respectively. These main weight losses, credited to the degradation of the polymeric backbone, are in agreement with the maximum degradation temperatures of other PHB [62,63] and PLA [34,64] grades reported in the literature. Apart from PLA 3100HP, the other matrices showed a small degradation step at 338 °C, 395 °C, and 456 °C for PHB P209E, PHB P226, and PLA 3D860, respectively, which were likely due to the degradation of the additives.

PHB-based composites have three different degradation steps: (i) a small weight loss attributed to water vaporization starting at approximately 100 °C, associated with the humidity of the fibers; (ii) a main weight loss step between 236 and 287 °C, related to the degradation of PHBs’ main chain; and (iii) the step related to the thermal degradation of cellulose between 335 and 350 °C (Figure S4c) [22,65]. Additionally, the weight losses associated with this last step match the different reinforcement loads of the fibers. These ternary degradation profiles have also been reported by other authors for PHB-based composites loaded with other natural fibers (e.g., wood fibers and flax) [22,66]. The maximum degradation temperatures of PHB P226-based composites were similar to those of the pristine matrix (around 280 °C), but for composites with PHB P209E (Figure 11A), the incorporation of high contents of cellulose fibers, e.g., 40 wt.% load, increased the maximum thermal degradation from 236 °C to over 280 °C. This increase proves that, although the compatibility of PHB with the micronized fibers is not excellent, as seen in the SEM micrographs, there is some degree of interfacial adhesion between them [35]. Even if increases in the thermal stability have been reported in other studies, as is the example of PHB reinforced with agave fibers [39], the effects of the fiber incorporation on the thermal stability of PHB-based composites are debatable, with most studies showing decreases on the stability upon incorporation of natural fibers such as piassava [67], flax [66] almond shell, or rice husk [68].

For composites with PLA, however, given the relatively good interfacial adhesion between the micronized fibers and these thermoplastic polymers, as well as the similar thermal stabilities of PLAs and micronized cellulose fibers, only a main weight loss can be observed on the thermograms in Figure 11. Regarding the composites with PLA 3D860 as the matrix, the incorporation of fibers led to an increase in the maximum degradation temperature of the material (Figure 11C). For instance, the addition of 40 wt.% of Cel355 raised the maximum degradation temperature by 11 °C from 336 °C to 347 °C, which can be justified by the lower maximum degradation temperature of PLA 3D860 (336 °C) in comparison with Cel355 (350 °C) (Figure S4c). On the contrary, because the maximum degradation temperature of PLA 3100HP (364 °C) was superior to that of Cel355, the gradual increase on the fiber load led to a slight reduction on the maximum degradation temperature of the composite (Figure 11D). In a similar research study, Espinach et al. [64] investigated the thermal properties of composites made of PLA reinforced with bleached kraft softwood and also concluded that the decrease in the thermal stability of the composites was due to the presence of a cellulosic filler with a lower thermal stability than the PLA. The existence of only one peak also strengthens the relatively good compatibility between PLA and cellulosic fibers.

Analogous to the composites with different fiber loads, composites based on PHBs reinforced with 40 wt.% of micronized fibers with different aspect ratios displayed the same ternary degradation profile and PLA-based composites only exhibited one major single degradation step. The similar degradation patterns and degradation temperatures observed in Figure S5 allow us to withdraw the conclusion that the aspect ratio of the micronized fibers does not influence the thermal stability of the composites. This conclusion corroborates the results obtained for the tensile, flexural, and impact mechanical properties, as well as the results from MFR in the sense that different aspect ratios of the micronized fibers gave origin to composites with identical properties.

## 3. Materials and Methods

### 3.1. Materials

Two poly(lactic acid) (PLA) pellet samples: Ingeo™ Biopolymer 3D860, with a melt flow rate (MFR) of 5–7 g·10 min^−1^ (210 °C, 2.16 kg), density of 1.22 g·cm^−3^, and relative viscosity of 4.0, and Ingeo™ Biopolymer 3100HP with an MFR of 24 g·10 min^−1^ (210 °C, 2.16 kg), density of 1.24, relative viscosity of 3.1, and molecular weight of 148 kDa [69], were supplied by NatureWorks (Minnnetonka, Minnesota, USA). Two commercial pellet grades of poly(hydroxybutyrate) (PHB): Biomer^®^ P209E, with an MFR of 10 g·10 min^−1^ (180 °C, 2.16 kg), density of 1.20 g·cm^−3^, and molecular weight of 0.6 × 10^6^ [70], and Biomer^®^ P226 with an MFR of 10 g·10 min^−1^ (180 °C, 5 kg), density of 1.25 g·cm^−3^, and number-average molecular weight of 22,200 ± 4500 [71], were purchased from Biomer Biopolyesters (Schwalbach, Germany). PHBs samples had 0–40 wt.% of a plasticizer and an unreported amount of a nucleating agent [27]. The chemical and crystalline structures of the thermoplastic matrices were confirmed through Fourier-transform infrared spectroscopy (FTIR) and X-ray diffraction, as shown in Figures S1 and S2, respectively.

Bleached Eucalyptus kraft pulp (BEKP) with a crystallinity index (CI) of 70.1% was kindly provided by The Navigator Company (Aveiro, Portugal). The micronized fibers were obtained from the same BEKP on a drum milling equipment (Pallman Fine grinding PS, Zweibrücken, Germany) armed with knives. The BEKP was fed into the cutting chamber and cut repeatedly between the rotor knifes and stator knifes against each other until the material could pass through the screen insert. Different sieve meshes were used to produce fibers with different aspect ratios, namely Cel60 (11.0), Cel200 (17.2), Cel355 (26.6), and Cel500 (28.9). The micrographs showing their fibrillar morphology as well as the length and width distributions are presented in Figure S3, and the average length, width, aspect ratio, and crystallinity indexes are shown in Table 1. The CI of BEPK and of all the microfibers (Figure S4b) were determined by X-ray diffraction based on the peak height method. A Phillips X’pert MDP diffractometer (PANalytical, The Netherlands) using CuKα radiation (λ = 1.541 Å) with a scan rate of 0.05° s^−1^ was used for this analysis [72].

### 3.2. Compounding and Processing of the Biocomposites

Composites with different percentage loads of Cel355 (micronized fibers with an intermediate aspect ratio) ranging from 10 to 40 wt.%, relative to the total weight of the composite, were compounded with the four distinct thermoplastic polymers: PHB P209E, PHB P226, PLA3D860, and PLA3100HP. Additionally, to evaluate the influence of the fibers aspect ratio, the four thermoplastic matrices were also compounded with the four micronized pulp fibers and the non-treated BEKP for a fixed reinforcement load of 40 wt.%. All composites were manufactured by melt-mixing in a Brabender W 30 EHT Plastograph EC mixer (Duisburg, Germany) with a total volume capacity of 30 cm^3^. The thermoplastic polymeric matrices and cellulose fibers were mixed for 15 min at a screw speed of 50 rpm at a temperature of 180 °C for PLA 3100HP and 170 °C for the remaining polymers.

Test specimens for the mechanical and water-uptake assays were prepared by injection molding in a Thermo Scientific Haake Minijet II (Waltham, MA, USA). For the PHB-based composites, the injection temperature was set at 175 °C, the mold temperature at 60–65 °C, injection pressure of 400 bar for 20 s, and post-injection pressure of 200 bar for 5 s. For the PLA-based composites, the injection temperature was 195 °C, with a mold temperature of 100–130 °C and injection pressure of 800 bar.

### 3.3. Characterization

The density of the polymeric matrices and composites were calculated by dividing the weight of the test specimens by their volume. At least five specimens (80 × 10 × 4 mm^3^) with a volume of 3.2 cm^3^ were weighted for each sample and both the mean and standard deviation calculated.

Scanning electron microscopy (SEM) analysis were performed on a FE-SEM Hitachi SU70-47 microscope (Hitachi High-Technologies Corporation, Tokyo, Japan), operated at 15.0 kV. The cross-section micrographs were obtained from the tensile test specimens after breaking. Prior to the analysis, all samples were coated with a carbon film.

Tensile and flexural assays were carried out on the universal testing machine Instron 5564 (Instron Corporation, Norwood, MA, USA). The tensile properties of at least six specimens were tested in accordance with the ISO-527-2 procedure (bar type3) at a velocity of 5 mm·min^−1^ using a 10 kN static load cell. For the flexural modulus, the three-point loading model was used according to ISO 178. Five specimens (80 × 10 × 4 mm^3^) were tested at a crosshead velocity of 5 mm·min^−1^ and at a length of span between supports of 64 mm using a 500 N static load cell. To measure the unnotched Charpy (edgewise) impact strength, a Ray Ran Universal Pendulum impact system (Ray-Ran Test Equipment Ltd., Nuneaton, UK), operating a pendulum of 4 J, was used according to ISO 179/1eU. The support span was set at 62 mm. Ten specimens with dimensions of 80 × 10 × 4 mm^3^ were tested for each sample and the average values were calculated.

The water-uptake capacity was assessed by immersing composite specimens (60 × 10 × 1 mm^3^) in water at room temperature during a period of 31 days. The weight of the samples was periodically assessed after removing the excess water with tissue paper. The water uptake (%) at time *t* was calculated according to Equation (1):

(1) $$Water\;uptake\;\left(\%\right)=\frac{\left(W_{t}-W_{0}\right)}{W_{0}}\times 100$$

where *W*_0_ is the specimen’s initial weight and *W_t_* is the weight of the specimens after the immersion time in grams. The mean and standard deviation were calculated for three replicates.

The melt flow rate of the different samples was evaluated using the Melt Flow Indexer Davenport (MFR-9) (Ametek, Denmark) operated at 190 °C for the PLA-based composites and at 175 °C for the PHB-based composites. The cut-off intervals were chosen according to the ASTM D1238 standard. At least five cut-offs for each sample were weighted and the melt flow rate was calculated as follows:

(2) $$MFR\;\left(g\cdot 10{\;\min}^{-1}\right)=\frac{600\times m}{t}\;$$

where *m* is the average mass of the cut-offs in grams and *t* is the cut-off time interval in seconds.

The thermal stability of the composites was evaluated with a SETSYS Setaram TGA analyzer (SETARAM Instrumentation, Lyon, France) equipped with a platinum cell. Approximately 10 mg of each sample was heated from room temperature to 800 °C at a constant rate of 10 °C·m^−1^ under a nitrogen atmosphere.

Statistical analysis of all the mechanical properties data was performed using the analysis of variance (ANOVA) and Tukey’s mean comparison test (OriginPro 9.6.5, OriginLab Corporation, Northampton, MA, USA) with the statistical significance established at *p* < 0.05.

## 4. Conclusions

Micronized cellulose fibers obtained from bleached Eucalyptus kraft pulp were investigated as reinforcements in composites with bio-based matrices (PLA and PHB). The influence of the fiber load and fiber aspect ratio on the performance of the composites was thoroughly studied.

The composites with micronized fibers displayed overall good homogeneity with no visible fiber agglomerates. Reinforcing the composites with increasing contents of micronized fibers significantly improved the Young’s modulus, tensile strength, and flexural modulus, while decreasing the elongation at break, strain at break, and the impact strength. The increase in the water uptake and the decrease of the melt flow rate were also consequences of the increasing reinforcement content. For the most part, the maximum degradation temperatures of the composites were slightly raised with the incorporation of the fibers, especially in composites based on PHB P209E and PLA 3D860.

For composites with PHBs, the water-uptake resistance, melt flowability, and mechanical performance, namely the tensile strength and both the Young’s and flexural moduli, were significantly improved when using micronized fibers rather than non-treated BEKP. However, the aspect ratio of the micronized fibers had little influence on the tensile, flexural, and impact properties, as well as on the thermal stability of the composites. In short, the overall results lead us to conclude that the micronization of the BEKP was an efficient and convenient method to further improve the performance of the composites, without the use of any hazardous solvents or chemicals. The mechanical and flow properties show the potential of the present fully sustainable composites as an alternative to the existing partially bio-based ones for injection molding applications intended for electronics, furniture, house appliances, and other applications.

## Figures and Tables

**Table 1:** Average dimensions and crystallinity indexes of the cellulose fibers used in this study.

Fiber Sample	Length (µm)	Width (µm)	Aspect Ratio	Crystallinity Index (%)
Cel60	149 ± 129	13.6 ± 5.4	11.0	54.1
Cel200	257 ± 170	14.9 ± 4.6	17.2	65.4
Cel355	332 ± 211	12.5 ± 5.4	26.6	64.6
Cel500	405 ± 203	14.4 ± 4.5	28.9	68.4
BEKP	770 ± 0.006	18.2 ± 0.1	42.3	70.7

## Data Availability

Not applicable.

## Supplementary Materials

The following are available online: FTIR-ATR spectra and X-ray diffractograms of the thermoplastic matrices; SEM micrographs and size (width and length) histograms of the micronized fibers; FTIR-ATR spectra, X-ray diffractograms, and TGA thermograms of the micronized fibers; density of the matrices and composites, and TGA thermograms of the composites reinforced with 40 wt.% fibers having different aspect ratios.

## Sample Availability

Samples of the compounds are not available from the authors.
