# Supplementary material for: Effect of the Micronization of Pulp Fibers on the Properties of Green Composites
Source: Molecules. 2021 Sep 15;26(18):5594. doi: 10.3390/molecules26185594 (PMC8468071; doi:10.3390/molecules26185594)
Supplement: Supplementary file 1 [file molecules-26-05594-s001.zip › molecules-1351695-supplementary.pdf]

molecules

Supplementary Material

Effect of the Micronization of Pulp Fibers on the Properties of Green Composites

Bruno F.A. Valente [1], Armando J.D. Silvestre [1], Carlos Pascoal Neto [2], Carla Vilela [1] and Carmen S.R. Freire [1,*]

[1] CICECO—Aveiro Institute of Materials, Department of Chemistry, University of Aveiro, Campus Universitário de Santiago, 3810-193 Aveiro, Portugal; bfav@ua.pt (B.F.A.V.); armsil@ua.pt (A.J.D.S.); cvilela@ua.pt (C.V.)
[2] RAIZ—Research Institute of Forest and Paper, The Navigator Company, Rua José Estevão, 3800-783 Eixo, Portugal; Carlos.Neto@thenavigatorcompany.com
* Correspondence: cfreire@ua.pt

Characterization of the Thermoplastic Matrices

Fourier transform infrared–attenuated total reflection (FTIR–ATR) spectra were recorded with a Perkin-Elmer FT–IR System Spectrum BX spectrophotometer (Perkin-Elmer Inc., Waltham, MA, USA) equipped with a single horizontal Golden Gate ATR cell over the range of 600–4000 cm^{-1} at a resolution of 4 cm^{-1} over 32 scans. The FTIR–ATR spectra of the two grades of PHB from Biomer and the two grades of PLA from Natureworks were used as matrices to manufacture the biocomposites shown in Figure S1. For the PLAs, the FTIR–ATR spectra showed the characteristic stretching frequencies at 1753 cm^{-1}, 2996 cm^{-1}, 2945 cm^{-1}, and 1081 cm^{-1} as assigned to C=O, CH_3 asymmetric, CH_3 symmetric, and C–O, respectively, and the characteristic bending frequencies of 1453 cm^{-1} and 1358 cm^{-1} as attributed to asymmetric and symmetric CH_3, respectively [1]. Similarly to the PLAs, the stretching vibration for both PHBs at 1719 cm^{-1} are also attributed to the ester carbonyl groups. The vibrations between 1000 cm^{-1} and 1300 cm^{-1} are allocated to the C–O of the ester groups, while the stretching vibrations at 2960 cm^{-1} and 2933 cm^{-1}, and the bending vibrations at 1378 cm^{-1} and 1455 cm^{-1}, correspond to the CH_3 groups [2].

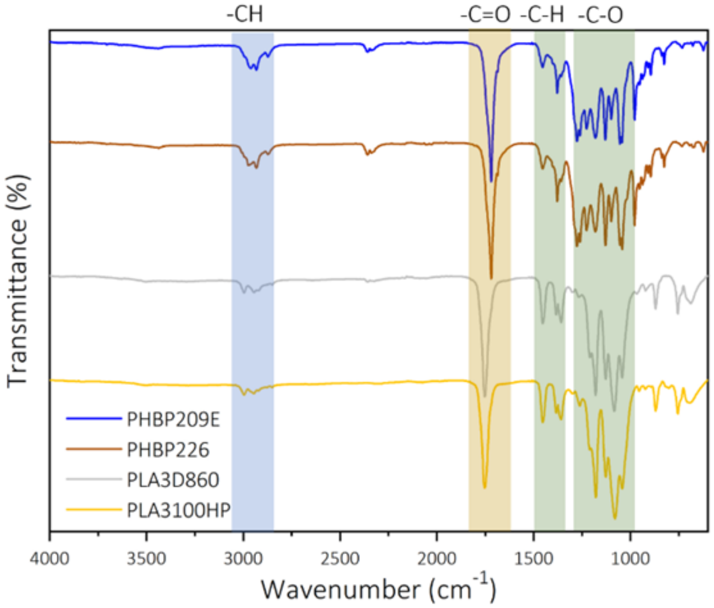

Figure S1. FTIR–ATR spectra of the different PLA and PHB matrices used in the present work.

X-ray diffraction (XRD) was performed on a Phillips X'pert MPD diffractometer (PANalytical, Netherlands) using CuKα radiation (λ=1.541 Å) with a scan rate of 0.05° s^{-1}. The XRD patterns were collected in reflection mode with the samples placed on a Si wafer (negligible background signal) for mechanical support and thus avoiding sample bending. Regarding the crystallinity of the polymers (Figure S2), both PHBs displayed the typical pattern reported in the literature with peaks at 2θ=13° and 17° assigned to (020) and (110) of an orthorhombic cell unit, and peaks at 21.5° and 22.5° attributed to (101) and (111) of α PHB crystals [3]. The small peak at 2θ=20° has been attributed to β-form crystals. Other peaks at 2θ= 26° (130) and 27° (040) have also been reported [3]. In addition to the reflections attributed to the PBH, the grade P226 exhibited some high intensity peaks at 2θ=9.5°, 19°, 26.7°, and 28.5°, which may have been caused by the presence of additives such as lubricants and nucleating agents. Moreover, the PLA 3D860 had a strong peak at 2θ=16.6° and three weaker peaks at 2θ=15°, 19°, and 22.5° are in accordance with results published by other authors [4]. For PLA 3100HP, only the peak at 2θ=16.6° was observed.

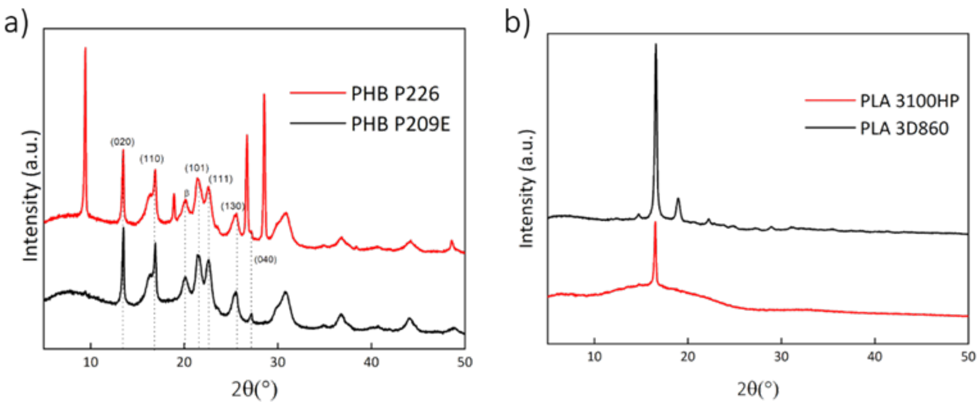

Figure S2. X-ray diffractograms of the polymeric matrices: a) PHBs and b) PLAs.

Characterization of the Micronized Fibers

The characterization of the micronized pulp fibers, i.e., micronized BEKP, by means of SEM, XRD, FTIR-ATR spectroscopy, and TGA, allowed for the evaluation of the fibers with respect to their morphology, crystallinity, chemical structure, and thermal stability. The length and width of at least 100 fibers for each sample were measured from SEM micrographs using the software ImageJ (version 1.51j8), as reported elsewhere [5]. The histograms in Figure S3 show that for every type of cellulose, there were fibers with lengths that ranged from a few micrometers to one millimeter. However, it is clear that the average length increased from Cel60 to Cel200, Cel355, and Cel500. Since the micronization did not have a significant influence on the fibers' widths, varying from 12.5 to 14.9 μm, the fiber aspect ratio followed the same order as the fiber length.

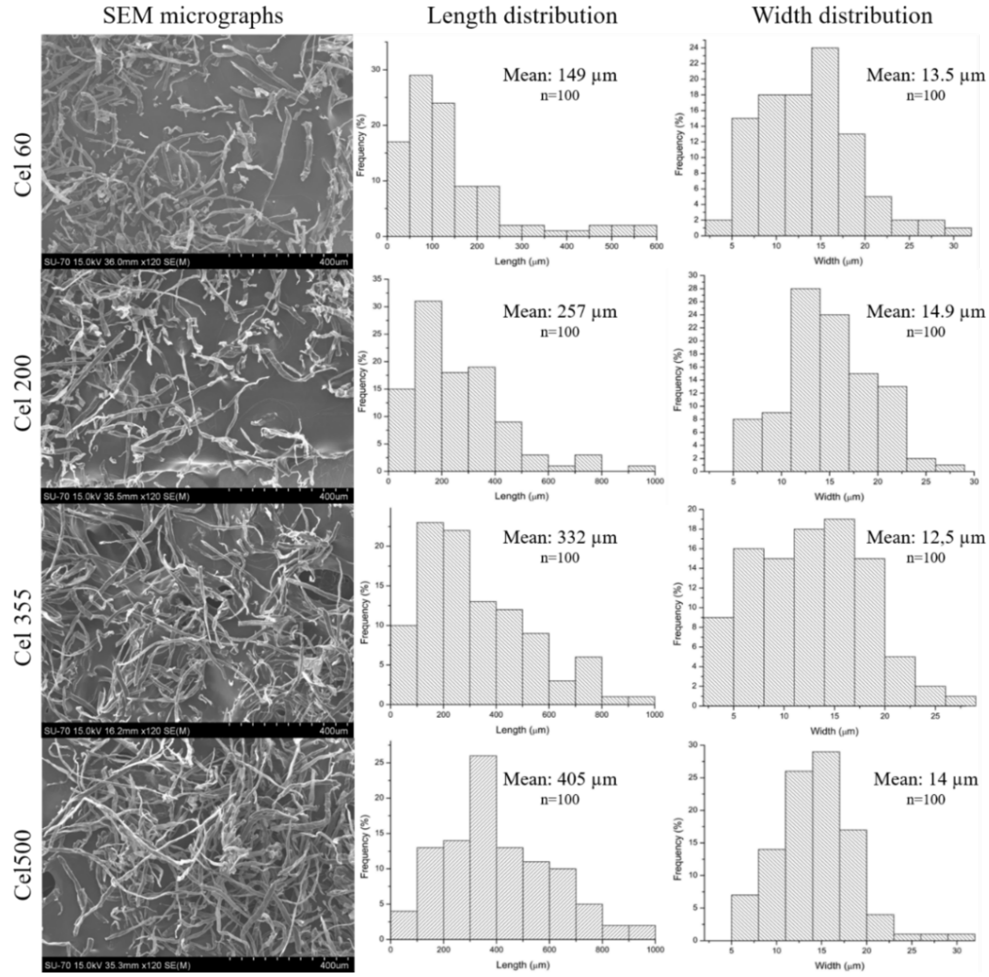

Figure S3. SEM micrographs and both length and width distributions of the different micronized pulp fibers.

The FTIR–ATR analyses of the micronized BEKP (Cel355) are represented in Figure S4a. The vibrations at 3200–3400 cm^{-1} (OH stretching), 2900 cm^{-1} (CH stretching), 1426–1430 cm^{-1} (CH_2 bending), 1350– 1355 cm^{-1} (CH bending), 1310 cm^{-1} (OH bending), 1025 cm^{-1} (CO stretching), and 900 cm^{-1} (CH bending) are according to the typical absorption vibrations of a cellulosic substrate [6].

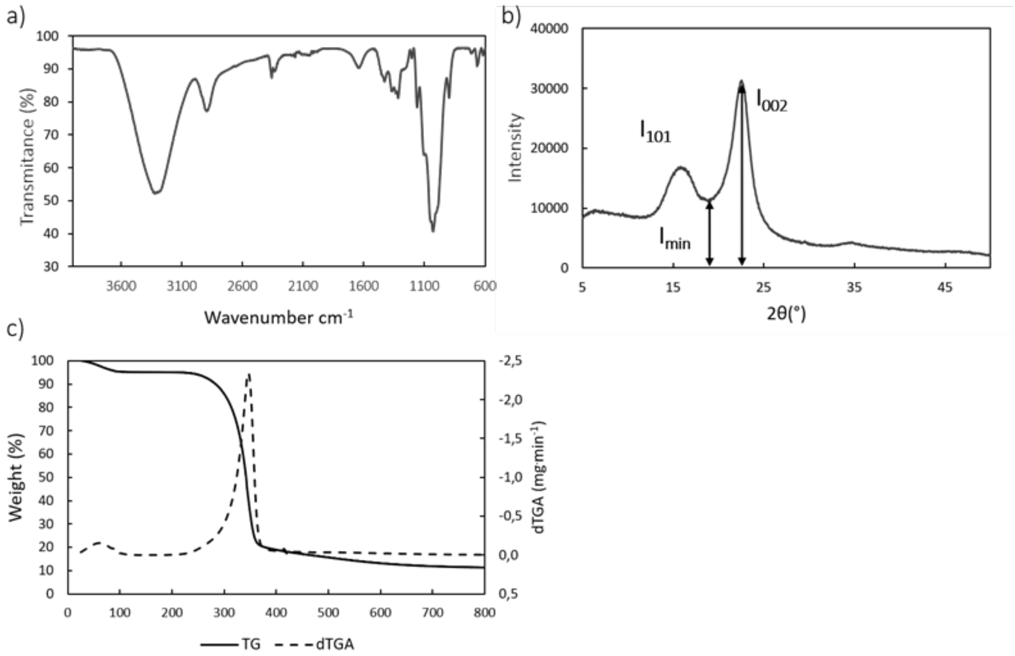

Figure S4. a) FTIR-ATR spectrum; b) XRD diffractogram; and c) TGA thermogram of Cel355.

The crystalline index (CI) of the different cellulose fibers was calculated by the peak height method from the ratio of the height of the 002 peak (I_{002}) and the height of the minimum (I_{min}) between the 002 and 101 peaks (Equation (S1)) [7].

$$CI\ (\%) = \frac{I_{002} - I_{min}}{I_{002}} \times 100 \quad \text{(S1)}$$

Copyright: © 2021 by the authors. Licensee MDPI, Basel, Switzerland. This article is an open access article distributed under the terms and conditions of the Creative Commons Attribution (CC BY) license (http://creativecommons.org/licenses/by/4.0/).

The micronized cellulose fibers showed the typical XRD pattern (Figure S4b) of cellulose I with the main diffraction peaks at about 2θ = 14-17° and 22.5°, which are usually assigned to the diffraction planes 101 and 002, respectively [6]. The crystallinity lowered with the decrease in the fiber length from 68.4% for Cel500 to 54.1% for Cel60. The CI for Cel355 and Cel200 rested in the middle with values of 64.6% and 65.4%, respectively. Compared to the bleached Eucalyptus kraft pulp CI (70.7%), all the micronized fibers displayed slightly inferior crystallinity values. Therefore, these results confirm that although the micronization did not affect the native crystalline structure of cellulose, the crystallinity index of the fibers was changed. Similar reductions in the crystallinity index of cellulosic fibers have been reported after other mechanical treatments (e.g., solid state shear milling) [8].

TGA thermograms of the micronized fibers (Cel355) are shown in Figure S4c. The thermal degradation profile is divided into two main weight-loss stages. The first, at around 100 °C, is related to water vaporization and the second to the degradation of cellulose, which has a maximum weight loss at around 348 °C, notably close to the values reported in the literature [9].

Density

The density of the composites was calculated by dividing the weight of the test specimens by their volume. At least five specimens (8×1×0.4cm) with a volume of 3.2 cm^3 were weighted for each sample and both the mean and standard deviation were calculated. As expected, an increase in the fiber load raised the density of the composite, given the superior density of cellulose in comparison with the thermoplastic polymers used in the present work. In contrast, the density of the composites with fibers having different aspect ratios remained relatively unchanged.

Table S1. Average density of the composites reinforced with different loads of Cel355 and of the composites reinforced with fibers having different aspect ratios for a load of 40 wt.%.

Sample	Density ($g{\cdot}cm^{-3}$)	Sample	Density ($g{\cdot}cm^{-3}$)
**PHB P209E**	**1.17 ± 0.02**	**PHB P209E 40wt.% Cel60**	1.31 ± 0.01
PHB P209E 10wt.% Cel355	1.21 ± 0.00	PHB P209E 40wt.% Cel200	1.31 ± 0.00
PHB P209E 30wt.% Cel355	1.28 ± 0.01	PHB P209E 40wt.% Cel500	1.31 ± 0.00
PHB P209E 40wt.% Cel355	1.31 ± 0.02	PHB P209E 40wt.% BEKP	1.32 ± 0.02
PHB P226	1.19 ± 0.03	PHB P226 40wt.% Cel60	1.35 ± 0.00
PHB P226 10wt.% Cel355	1.24 ± 0.00	PHB P226 40wt.% Cel200	1.34 ± 0.00
PHB P226 30wt.% Cel355	1.29 ± 0.02	PHB P226 40wt.% Cel500	1.34 ± 0.00
PHB P226 40wt.% Cel355	1.34 ± 0.01	PHB P226 40wt.% BEKP	1.34 ± 0.01
PLA 3D860	1.22 ± 0.00	PLA 3D860 40wt.% Cel60	1.35 ± 0.00
PLA 3D860 10wt.% Cel355	1.25 ± 0.00	PLA 3D860 40wt.% Cel200	1.34 ± 0.02
PLA 3D860 30wt.% Cel355	1.31 ± 0.00	PLA 3D860 40wt.% Cel500	1.34 ± 0.00
PLA 3D860 40wt.% Cel355	1.35 ± 0.00	PLA 3D860 40wt.% BEKP	1.34 ± 0.00
PLA 3100HP	1.24 ± 0.02	PLA 3100HP 40wt.% Cel60	1.37 ± 0.00
PLA 3100HP 10wt.% Cel355	1.26 ± 0.03	PLA 3100HP 40wt.% Cel200	1.37 ± 0.00
PLA 3100HP 30wt.% Cel355	1.33 ± 0.01	PLA 3100HP 40wt.% Cel500	1.37 ± 0.00
PLA 3100HP 40wt.% Cel355	1.38 ± 0.00	PLA 3100HP 40wt.% BEKP	1.36 ± 0.00

Thermogravimetric Analysis

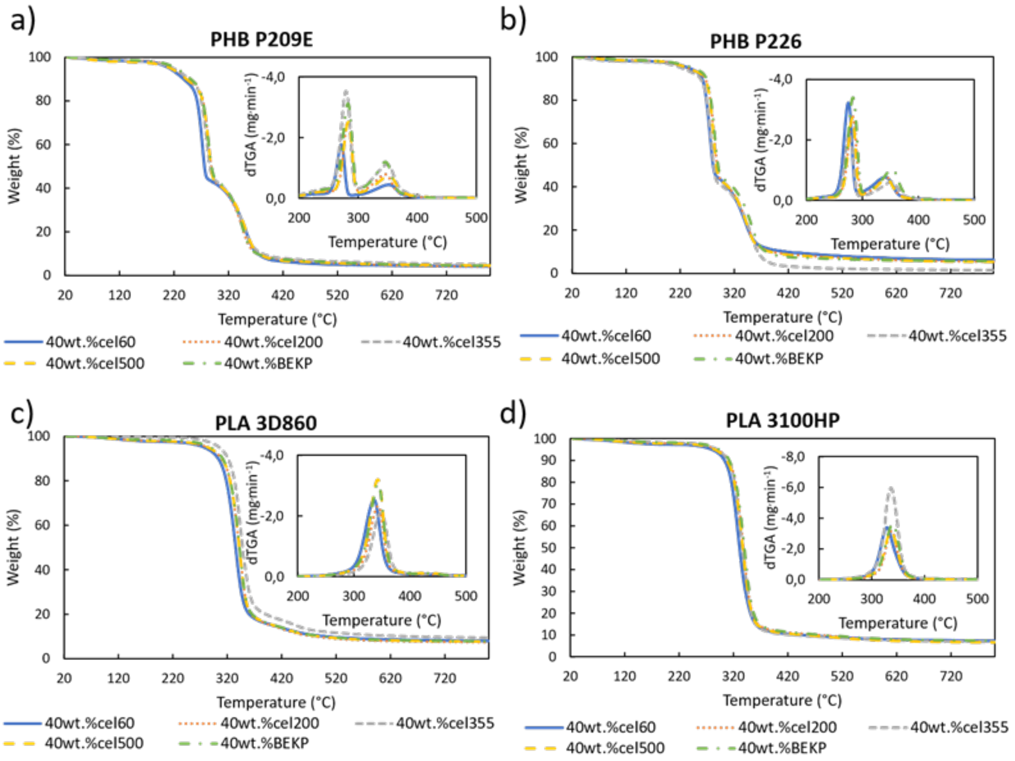

Figure S5. Thermogravimetric and derivative (dTGA) curves of the composites reinforced with micronized fibers having different aspect ratios.

References

1. Chieng, B.W.; Ibrahim, N.A.; Yunus, W.M.Z.W.; Hussein, M.Z. Poly(lactic acid)/poly(ethylene glycol) polymer nanocomposites: Effects of graphene nanoplatelets. *Polymers (Basel).* **2014**, *6*, 93–104, doi:10.3390/polym6010093.
2. Ramezani, M.; Amoozegar, M.A.; Ventosa, A. Screening and comparative assay of poly-hydroxyalkanoates produced by bacteria isolated from the Gavkhooni Wetland in Iran and evaluation of poly-β-hydroxybutyrate production by halotolerant bacterium Oceanimonas sp. GK1. *Ann. Microbiol.* **2015**, *65*, 517–526, doi:10.1007/s13213-014-0887-y.
3. Mottina, A.C.; Ayres, E.; Orefice, R.L.; Câmara, J.J.D. What changes in poly(3-hydroxybutyrate) (PHB) when processed as electrospun nanofibers or thermo-compression molded film? *Mater. Res.* **2016**, *19*, 57–66, doi:10.1590/1980-5373-MR-2015-0280.
4. Kumar, R.; Kumari, S.; Rai, B.; Das, R.; Kumar, G. Effect of nano-cellulosic fiber on mechanical and barrier properties of polylactic acid (PLA) green nanocomposite film. *Mater. Res. Express* **2019**, *6*, 125108, doi:10.1088/2053-1591/ab5755.
5. Tanpichai, S.; Witayakran, S. All-cellulose composites from pineapple leaf microfibers: Structural, thermal, and mechanical properties. *Polym. Compos.* **2018**, *39*, 895–903, doi:10.1002/pc.24015.
6. Foster, E.J.; Moon, R.J.; Agarwal, U.P.; Bortner, M.J.; Bras, J.; Camarero-Espinosa, S.; Chan, K.J.; Clift, M.J.D.; Cranston, E.D.; Eichhorn, S.J.; et al. Current characterization methods for cellulose nanomaterials. *Chem. Soc. Rev.* **2018**, *47*, 2609–2679, doi:10.1039/C6CS00895J.
7. Park, S.; Baker, J.O.; Himmel, M.E.; Parilla, P.A.; Johnson, D.K. Cellulose crystallinity index: Measurement techniques and their impact on interpreting cellulase performance. *Biotechnol. Biofuels* **2010**, *3*, 10, doi:10.1186/1754-6834-3-10.
8. Yang, S.; Bai, S.; Wang, Q. Sustainable packaging biocomposites from polylactic acid and wheat straw: Enhanced physical performance by solid state shear milling process. *Compos. Sci. Technol.* **2018**, *158*, 34–42, doi:10.1016/j.compscitech.2017.12.026.
9. Yeng, L.C.; Wahit, M.U.; Othman, N. Thermal and flexural properties of regenerated cellulose(RC)/poly(3-hydroxybutyrate)(PHB)biocomposites. *J. Teknol.* **2015**, *75*, 107–112, doi:10.11113/jt.v75.5338.
